# Rectal diffuse large B-cell lymphoma misdiagnosed as bleeding cancer in an elderly patient

**DOI:** 10.1007/s12672-026-04551-x

**Published:** 2026-01-31

**Authors:** Yuchun Zhong, Qiansen Zhang, Yujie Fu, Jiusi Liu, Linhui Leng, Wei Xu

**Affiliations:** 1 Department of General Surgery, The Second Affiliated Hospital of Nanchang University, Nanchang, Jiangxi China; 2 Nanchang University, Nanchang, Jiangxi China; 3 The Institute of Translational Medicine, The Second Affiliated Hospital of Nanchang University, Nanchang University, Nanchang, Jiangxi China; 4 Department of Gastrointestinal Surgery, Ji’an Central People’s Hospital, Ji’an, Jiangxi China; 5 The General Surgery Department of the First People’s Hospital of xiushui County, Jiujiang, Jiangxi China

**Keywords:** Case report, Rectal diffuse large B-cell lymphoma, Hematochezia, Diagnostic error

## Abstract

**Background:**

Primary rectal diffuse large B-cell lymphoma (DLBCL) is a rare malignancy that often closely mimics more common rectal pathologies such as adenocarcinoma or inflammatory bowel disease. This resemblance poses considerable diagnostic difficulty, and misdiagnosis may result in delayed treatment and unnecessary procedures, ultimately affecting patient outcomes.

**Case presentation:**

An 87-year-old female presented with intermittent hematochezia that acutely worsened two hours before admission. Physical examination identified a rectal mass located 5 cm from the anal verge. Pelvic imaging and colonoscopy were suggestive of hemorrhagic adenocarcinoma. Given the patient’s advanced age, significant anemia, and elevated bleeding risk, a multidisciplinary team decided against preoperative biopsy. Instead, transanal minimally invasive surgery was carried out for local disease control. Final histopathological analysis of the resection specimen revealed DLBCL, germinal center B-cell-like subtype. Following surgery, the patient was referred to hematology for guideline-directed chemotherapy and remained free of recurrence during follow-up.

**Conclusion:**

This case highlights that rectal DLBCL can present with hematochezia as the sole symptom and may be mistaken for rectal adenocarcinoma, particularly in elderly patients. The divergence between the preoperative clinical impression and the postoperative pathological diagnosis emphasizes both the diagnostic challenge and the tailored therapeutic approach required for this rare condition. A high index of suspicion for uncommon malignancies is crucial to ensure appropriate clinical management.

## Introduction

Extranodal lymphomas, such as primary gastric and colorectal mucosa-associated lymphoid tissue (MALT) lymphoma and primary central nervous system lymphoma (PCNSL), account for approximately 30% of cases [1, 2]. Molecular characteristics vary depending on the specific subtype. For instance, in Germinal Center B-Cell like Diffuse Large B-Cell Lymphoma (GCB-DLBCL), as represented in this case, germinal center markers such as CD10 and Bcl-6 serve as key distinguishing features compared to marginal zone B-cell lymphoma (MZBL) [2]. DLBCL is a relatively common, highly aggressive malignancy of the hematolymphoid system, characterized by an age-standardized incidence rate of approximately 0.72 per 10,000 person-years [3]. Its incidence increases with advancing age, predominantly affecting elderly populations. However, primary occurrence in the rectum is exceedingly rare [4]. And the clinical presentation of rectal DLBCL often resembles that of more common rectal pathologies, such as adenocarcinoma or inflammatory bowel disease, further adding to the difficulty of differentiation [5, 6]. This misdiagnosis may delay appropriate therapeutic intervention and may lead to unnecessary medical procedures that ultimately affect patient outcomes [7]. Amid global demographic aging, we present this rare case to enhance clinicians’ recognition of uncommon pathologies in geriatric patients, ultimately contributing to improved diagnostic accuracy and therapeutic outcomes for rare diseases.

## Case presentation

**Fig. 1:**
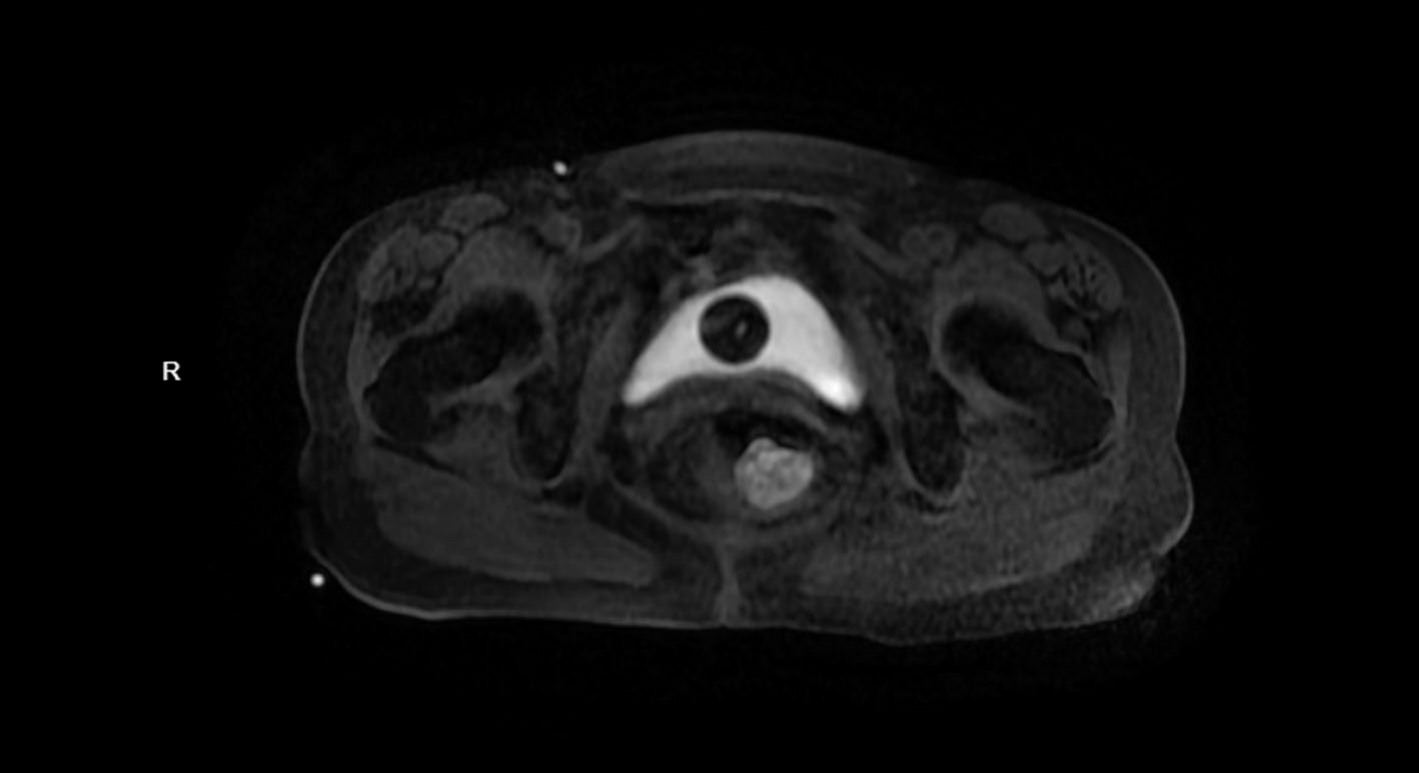
Imaging studies: Pelvic magnetic resonance imaging (MRI) with contrast enhancement revealed marked thickening of the right rectal wall, with a protuberant nodular lesion measuring approximately 32 × 27 mm

An 87-year-old female patient was admitted to our department from the emergency room with recurrent hematochezia (rectal bleeding) persisting for over half a month, which had acutely worsened over the preceding 2 h. The stool was bright red in color with a loose/pasty consistency, and the most recent episode was estimated at approximately 50 ml. The patient reported associated symptoms including dizziness, fatigue, and palpitations, but denied abdominal pain, distension, fever, nausea, or vomiting. Emergency digital rectal examination performed in the knee-chest position revealed a palpable mass on the right rectal wall. The mass was moderately firm in consistency, encircled approximately one-quarter of the rectal lumen, exhibited moderate mobility, and had relatively distinct borders. Blood staining the examination glove was noted upon withdrawal of the finger. Urgent complete blood count revealed: Hemoglobin: 93 g/L (Reference range: 115–150 g/L) (Table 1). Findings of pelvic magnetic resonance imaging (MRI) non-contrast and contrast-enhanced scan: Marked thickening of the right rectal wall with a protruding nodular shadow, measuring approximately 32 × 27 mm, showing isointense signal on T1-weighted images and moderately hyperintense signal on T2-weighted images. The lesion appears hyperintense on DWI and demonstrates significant enhancement after contrast administration, with blurring of the surrounding fat planes. Thus, imaging findings suggest a space-occupying lesion in the right rectal wall, consistent with changes of rectal carcinoma (Fig. 1). Based on the patient’s symptoms, physical examination findings, laboratory results, and imaging findings, a preliminary diagnosis of rectal carcinoma was considered.

Past medical history included hypertension and cerebral infarction. The patient was on long-term therapy with nifedipine and aspirin tablets. She denied any history of other gastrointestinal inflammatory or neoplastic diseases, significant personal or family medical history, or relevant travel exposure.

### Relevant medical laboratory examination

On admission, the patient’s vital signs were stable: body temperature 36.5 °C, blood pressure 164/66 mmHg, and pulse rate 88 beats per minute. Physical examination revealed no significant abnormalities in the head, neck, skin, mucous membranes, or superficial lymph nodes. The abdomen was soft without tenderness, rebound tenderness, muscle guarding or palpable masses.

### Laboratory investigations

Blood tests revealed: hemoglobin 93 g/L (Reference range: 115–150 g/L); Hematocrit (Hct): 29.0% (Reference range: 35–45%); Mean corpuscular volume (MCV): 65.6 fL (Reference range: 82–100 fL); indicating mild anemia. Other blood parameters were normal. All Tumor markers within normal reference ranges (Table 1).

**Table 1:**
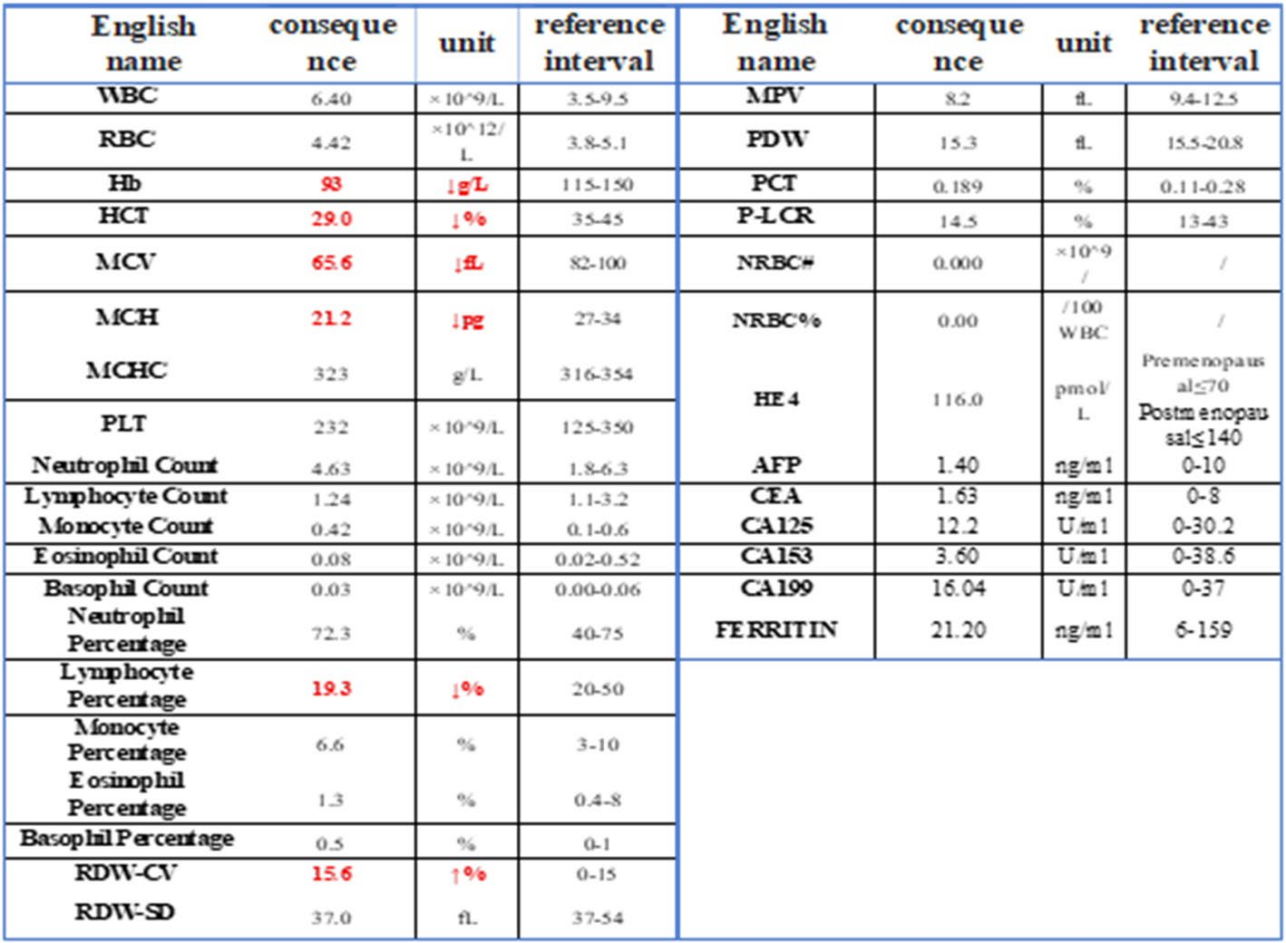
Results of emergency blood routine and tumor markers. Red indicates abnormality

### Diagnosis and treatment

**Fig. 2:**
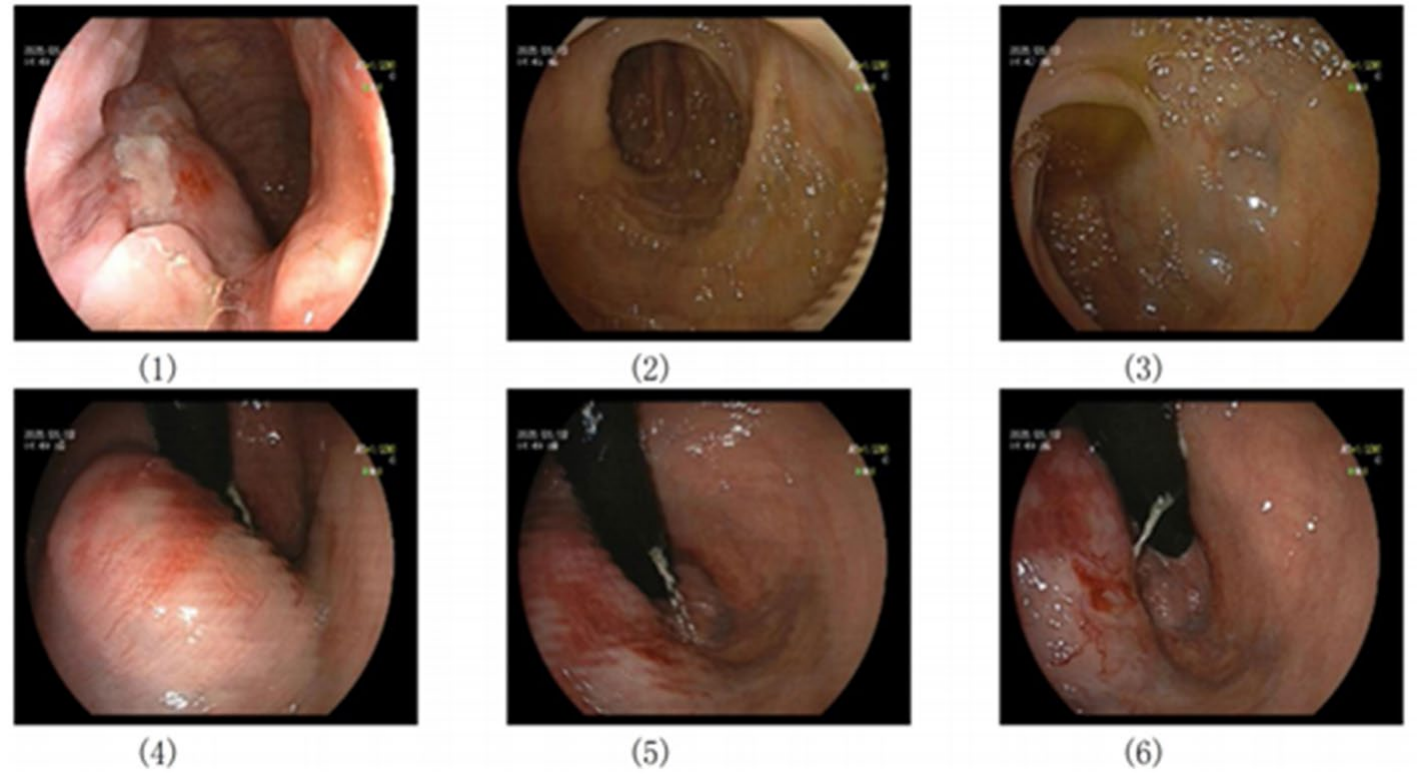
Colonoscopic Image of the tumor

To further elucidate the diagnosis, endoscopic examination was pursued. Endoscopy revealed an irregularly elevated mass approximately 5 cm from the anal verge. The lesion exhibited relatively distinct borders, an uneven surface with irregular contours, and multiple scattered bleeding points. The mass occupied approximately one-quarter of the rectal lumen circumference. The patient presented with persistent hematochezia after admission, which responded poorly to initial pharmacologic and endoscopic hemostatic measures. Furthermore, the patient was on long-term aspirin therapy for antiplatelet aggregation due to a previous cerebral infarction. Under these circumstances, although coagulation screening tests showed no significant abnormalities and hemodynamic parameters remained stable, performing an endoscopic biopsy still carried a considerable risk of secondary bleeding. Considering the patient’s advanced age, a history of cerebral infarction that may be associated with increased vascular fragility, and reduced tolerance to blood loss, the multidisciplinary team agreed that the risk of performing a biopsy for histologic diagnosis at this time outweighed its potential benefit. Therefore, the clinical decision was made to prioritize emergent hemostasis and systemic stabilization over proceeding with a biopsy (Fig. 2).

Based on the aforementioned diagnostic findings and the significant risk of hemorrhage, emergent transanal minimally invasive surgery was performed after comprehensive discussion with the patient and her family.

### Histopathological and immunohistochemical analysis

**Fig. 3:**
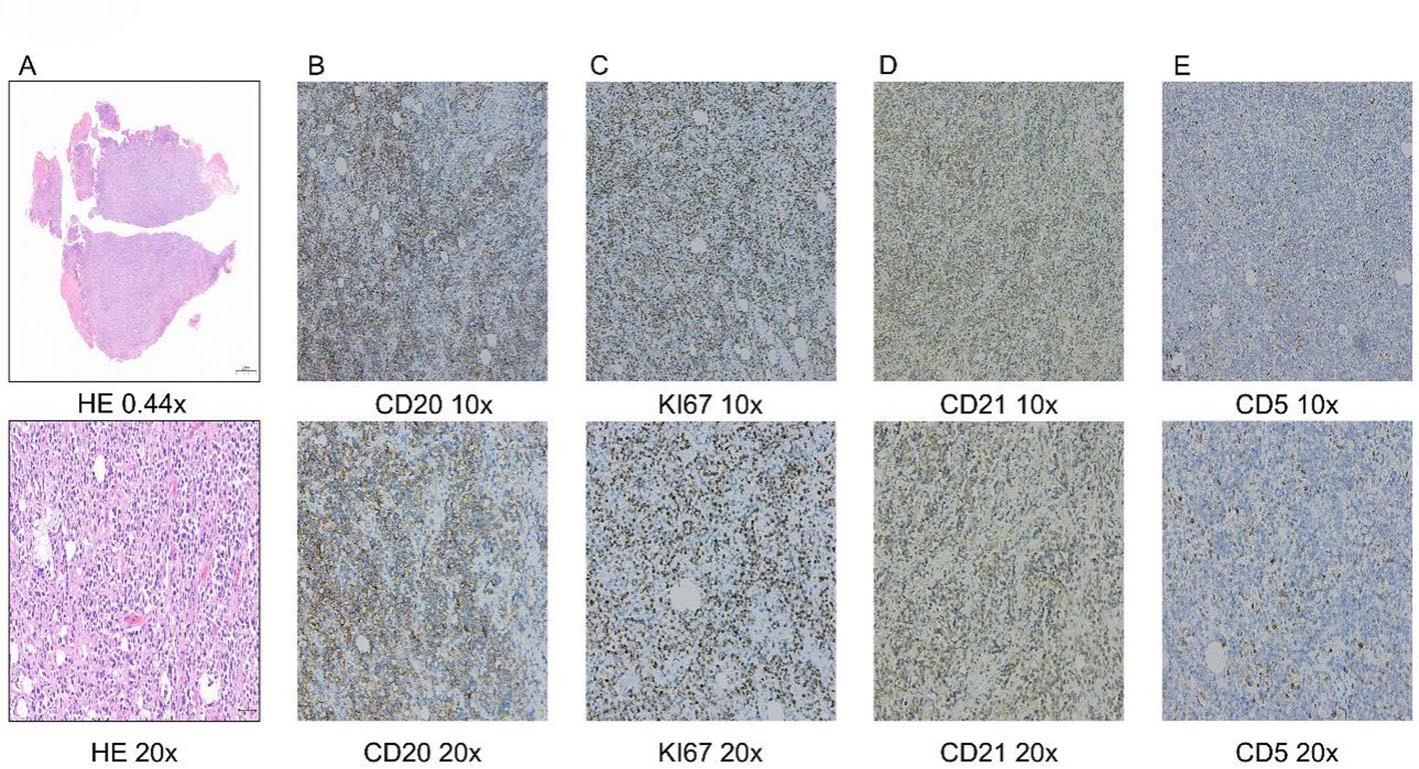
Histopathology and Immunohistochemical Staining.Immunohistochemical Analysis: CD20: Diffusely positive (+);Ki-67: Positive (+), approximately 95% (indicating high proliferative index); CD21: Highlighted disrupted follicular dendritic cell meshworks; CD5: Positive (+) in background T-cells (T-cell staining noted);** A**–**E** Row 1: 10x (HE 0.44x); Row 2: 20x

**Fig. 4:**
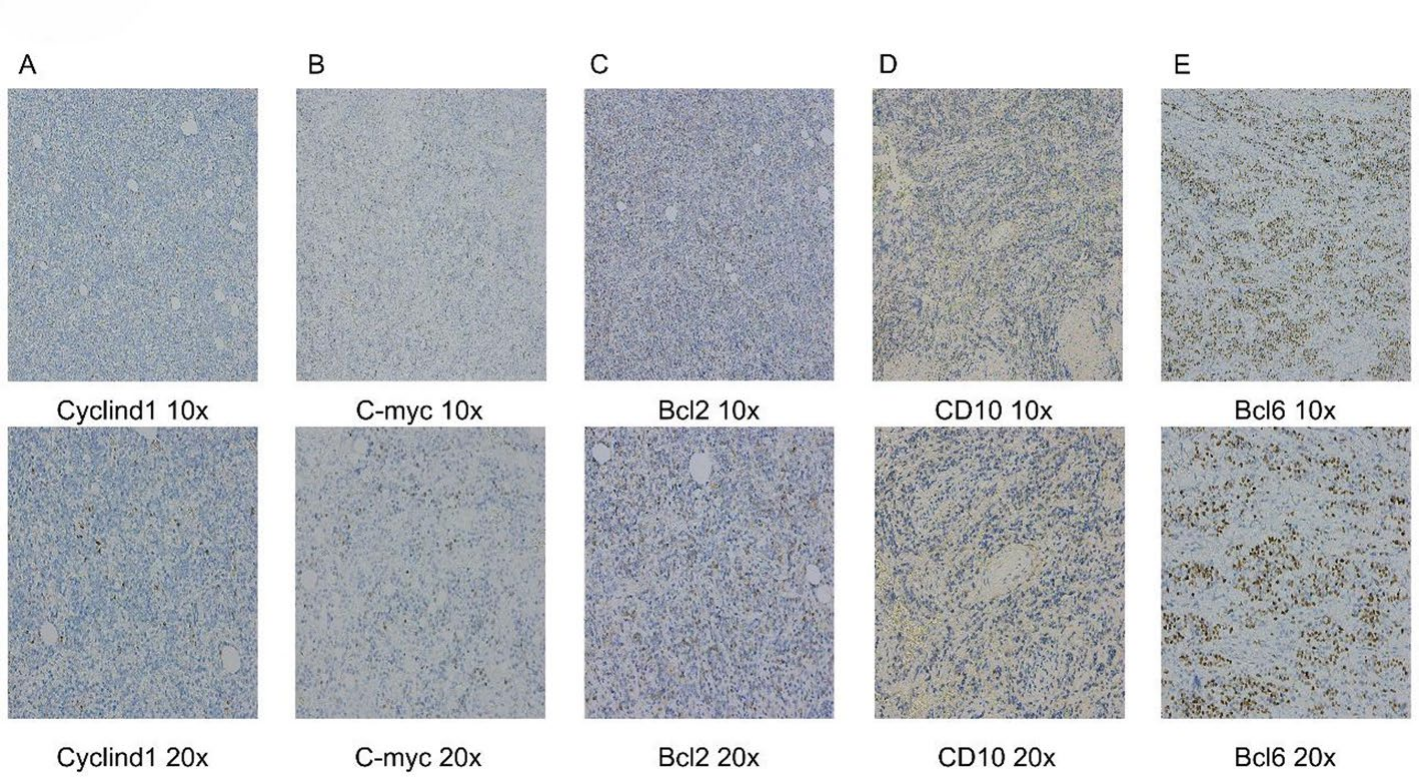
Histopathology and Immunohistochemical Staining.Immunohistochemical Analysis: Cyclin D1: Negative (-);C-myc: Positive (+), approximately 10% ;Bcl-2: Positive (+), approximately 40%; CD10: Diffusely positive (+); Bcl-6: Positive (+), approximately 70%;** A**–**E** Row 1: 10x ; Row 2: 20x

However, definitive histopathological examination of the resected tumor tissue revealed primary rectal diffuse large B-cell lymphoma (DLBCL) (Figs. 3 and 4). HE staining revealed tumor tissue exhibiting a diffuse sheet-like growth pattern. The tumor cells were large to intermediate in size, with large nuclei displaying hyperchromatic or pale, vesicular chromatin. Prominent nucleoli were evident in the majority of cells, and abundant karyorrhexis (nuclear fragmentation) was observed. The stroma showed vascular proliferation, with some vessels exhibiting dilatation and congestion. According to the classification of DLBCL reported in the literature [8], Combined with the definitive immunohistochemical analysis of CD20, KI67, CD21, CD5, Cyclin D1, C-myc, Bcl-2, CD10, and Bcl-6 (Table 2), the tumor tissue was confirmed as rectal diffuse large B-cell lymphoma (DLBCL) of the germinal center B-cell-like (GCB) subtype (Fig. 3).

**Table 2:** Results of immunohistochemical staining

IHC	HE	CD20	KI67	CD21	CD5
Results	+	+	+(95%)	+	+(Tcell)

IHC	Cyclind1	C-myc	Bcl2	CD10	Bcl6
Results	-	+(10%)	+(40%)	+	+(70%)

### Follow-up

Following surgery, the patient was referred to the Department of Medical Oncology and received systemic chemotherapy with the R-CHOP regimen (rituximab plus cyclophosphamide, doxorubicin, vincristine, and prednisone) for a planned 4 cycles. The treatment was well-tolerated with no grade 3 or higher adverse events reported. During subsequent regular follow-up, no signs of recurrence have been observed.

## Discussion

### Clinical presentation and diagnostic challenges of rectal DLBCL

Primary gastrointestinal lymphoma represents a rare malignancy; however, its incidence has been steadily increasing [9]. Within this spectrum, the rectal subtype of primary DLBCL constitutes less than 0.2% of all colorectal malignancies [10, 11]. The rectal DLBCL are typically non-specific, commonly including abdominal pain, weight loss, rectal bleeding (hematochezia), and altered bowel habits. These symptoms lack specificity and can easily mimic more common gastrointestinal disorders [12–14]. Additionally, due to this non-specific presentation, patients may initially report systemic signssuch as anemia, fever, and anorexia [12]. Patient age and gender may further influence clinical manifestations, thereby posing additional challenges to timely and accurate diagnosis. For example, the report by Ayesha Nusrat further indicates that the incidence of malignant tumors is higher in boys than in girls, and that non‑Hodgkin lymphoma (NHL) originating from the small and large intestines is associated with better overall survival compared to gastric NHL. Additionally, the types of NHL frequently observed vary across different age groups [15]. In comparison with the present case, studies by Wei Ding, Nao Imuta, and others on DLBCL have more comprehensively revealed the complexity of this disease. Existing reports on DLBCL often describe its coexistence with other types of lymphoma or solid intestinal tumors, underscoring the necessity of a comprehensive diagnostic approach once intestinal DLBCL is suspected [11, 16]. This diagnostic ambiguity often delays therapeutic intervention. In this case, the patient presented with acutely exacerbated hematochezia and anemia refractory to initial symptomatic management. Consequently, clinicians should maintain high vigilance and consider lymphoma in the differential diagnosis when encountering such symptoms [11]. In this case, the patient manifested persistent rectal bleeding with acute exacerbation, which aligns with the reported cases mentioned above.

The symptomatic overlap between rectal DLBCL and other rectal diseases represents a major diagnostic hurdle. Its clinical features may closely resemble those of rectal polyps, ulcerative colitis, and rectal adenocarcinoma, which notably complicates precise diagnostic differentiation [12, 13, 17]. Notably, in this case, contrast-enhanced MRI and visual endoscopic assessment initially suggested rectal adenocarcinoma. The endoscopically observed active tumor bleeding, combined with the patient’s advanced age, anemia, and heightened procedural bleeding risk, precluded biopsy prior to intervention.

### The treatment paradigms for rectal DLBCL

Systemic immunochemotherapy (R-CHOP regimen) remains the cornerstone primary treatment for rectal diffuse large B-cell lymphoma. However, current evidence demonstrates that combination therapy integrating surgical intervention and chemotherapy may represents the more effective strategy for improving outcomes in patients with small intestinal or colorectal DLBCL [12, 18–20]. In addition, in addition to differences in treatment modalities that may lead to differences in DLBCL efficacy, the study of Georgian Halcu et al. also showed that patients with high Ki-67 had faster disease progression and shorter average survival. In this case, the Ki-67 was as high as 95%, so it needs to be vigilant [21]. Furthermore, challenges in obtaining definitive diagnostic confirmation prior to intervention and the high incidence of presenting complications (such as obstruction, perforation, or bleeding) may contribute to the initially high resection rates observed in these patients [22]. This case similarly demonstrated refractory hematochezia unresponsive to conservative interventions alongside a high preoperative suspicion of rectal adenocarcinoma that could not be definitively confirmed prior to surgical intervention.

The transanal minimally invasive surgery combined with postoperative adjuvant therapy effectively mitigated ongoing bleeding risks and avoided the physical trauma associated with abdominoperineal resection in this elderly patient. Both the patient and family members expressed strong appreciation for our treatment strategy during pre- and postoperative communications.

Consequently, this case report aims to underscore to clinicians the critical importance of recognizing both the extreme rarity of rectal DLBCL and the diagnostic c complexities arising from its complicating presentations.

## Conclusion

This case highlights hematochezia as the cardinal manifestation of rectal DLBCL and underscores the critical importance of definitive histopathological confirmation in elderly patients with unexplained lower gastrointestinal bleeding to mitigate diagnostic delays. However, the extreme rarity of rectal DLBCL (< 0.2% of colorectal malignancies), its mimicry of adenocarcinoma on conventional investigations (digital rectal examination, endoscopy, and imaging), and therapeutic dilemmas arising from acute complications continue to impede optimal management. To improve the guarded prognosis of this aggressive malignancy, implementing structured preoperative diagnostic protocols and evidence-based multimodal therapeutic strategies remains imperative.

## Data Availability

All data generated or analysed during this study are included in this published article and its supplementary information files.

## References

[1] Yang H, Xun Y, Ke C, Tateishi K, You H. Extranodal lymphoma: pathogenesis, diagnosis and treatment. Mol Biomed. 2023;4:29. 37718386 10.1186/s43556-023-00141-3 PMC10505605

[2] Fiorentino V, Pizzimenti C, Pierconti F, Lentini M, Ieni A, Caffo M, Angileri F, Tuccari G, Fadda G, Martini M, Larocca LM. Unusual localization and clinical presentation of primary central nervous system extranodal marginal zone B–cell lymphoma: A case report. Oncol Lett. 2023;26:408. 37600340 10.3892/ol.2023.13994 PMC10436157

[3] Wang SS. Epidemiology and etiology of diffuse large B-cell lymphoma. Semin Hematol. 2023;60:255–66. 38242772 10.1053/j.seminhematol.2023.11.004 PMC10962251

[4] Maguire LH, Hawkins AT. Surgical resection for primary rectal lymphoma: support for local excision? J Surg Res. 2019;244:189–95. 31299435 10.1016/j.jss.2019.06.034 PMC6815698

[5] Suzuki T, Iwamoto K, Nozaki R, Saiki Y, Tanaka M, Fukunaga M, Yamada K. Diffuse large B-cell lymphoma originating from the rectum and diagnosed after rectal perforation during the treatment of ulcerative colitis: a case report. BMC Surg. 2021;21:50. 33478454 10.1186/s12893-021-01060-2 PMC7819197

[6] Lai Q, Zhao Y, Yan H, Peng H. Advances in diagnosis, treatment and prognostic factors of Gastrointestinal DLBCL. Leuk Res. 2023;135:107406. 37944240 10.1016/j.leukres.2023.107406

[7] Zanelli M, Sanguedolce F, Zizzo M, Palicelli A, Pellegrini D, Farinacci S, Soriano A, Froio E, Cormio L, Carrieri G, et al. Primary diffuse large B-Cell lymphoma of the urinary bladder: update on a rare disease and potential diagnostic pitfalls. Curr Oncol. 2022;29:956–68. 35200580 10.3390/curroncol29020081 PMC8870454

[8] Shaffer AL 3rd, Young RM, Staudt LM. Pathogenesis of human B cell lymphomas. Annu Rev Immunol. 2012;30:565–610. 22224767 10.1146/annurev-immunol-020711-075027 PMC7478144

[9] Alvarez-Lesmes J, Chapman JR, Cassidy D, Zhou Y, Garcia-Buitrago M, Montgomery EA, Lossos IS, Sussman D, Poveda J. Gastrointestinal tract lymphomas. Arch Pathol Lab Med. 2021;145:1585–96. 33836528 10.5858/arpa.2020-0661-RA

[10] Manzar GS, Cha EE, Corrigan KL, Yoder AK, Schrank BR, Nasr LF, Chihara D, Castillo LM, Nair R, Jain P, et al. Outcomes and toxicities in patients with diffuse-large B cell lymphoma involving the Gastrointestinal tract and digestive organs. Front Oncol. 2024;14:1447020. 39324011 10.3389/fonc.2024.1447020 PMC11422352

[11] Ding W, Luo H, Wang Y. Synchronous multiple primary cancers involving rectal cancer and diffuse large B-cell lymphoma of the right colon: A case report. Cureus. 2025;17:e83921. 40502867 10.7759/cureus.83921 PMC12151806

[12] Chen X, Wang J, Liu Y, Lin S, Shen J, Yin Y, Wang Y. Primary intestinal diffuse large B-cell lymphoma: novel insights and clinical perception. Front Oncol. 2024;14:1404298. 39211552 10.3389/fonc.2024.1404298 PMC11357906

[13] Kiamos A, Streit SG, Karan A, Boldig K, Attarha BO, Kahn Z, Omman R, Schey R, Gharia B. Unusual instance of primary diffuse large B-cell lymphoma of the colon. Cureus. 2023;15:e36083. 37065294 10.7759/cureus.36083 PMC10095599

[14] Thuraisingam A, Jaffry K, Papa B. Atypical presentation of diffuse large B-Cell lymphoma of the caecum in a young patient: A case report. Cureus. 2025;17:e86150. 40671973 10.7759/cureus.86150 PMC12266697

[15] Wu P, Zhu D, Lou Y, Wang X. Clinical characteristics and prognostic factors for primary pediatric and adolescent Non-Hodgkin lymphomas of the Gastrointestinal tract: a population-based study. World J Surg Oncol. 2023;21:353. 37968641 10.1186/s12957-023-03238-9 PMC10647069

[16] Imuta N, Miyai K, Tsuchiya M, Saito M, Sone T, Kobayashi S, Ogata S, Kimura F, Matsukuma S. Concurrent intestinal plasmablastic lymphoma and diffuse large B-cell lymphoma with a clonal relationship: a case report and literature review. J Pathol Transl Med. 2024;58:191–7. 38910357 10.4132/jptm.2024.05.14 PMC11261169

[17] Lu XH, Yu YJ, Tan SY, Ding YJ. Primary small intestinal diffuse large B-cell lymphoma masquerading as crohn’s disease: A case report. Chin Med J (Engl). 2017;130:2138–9. 28836567 10.4103/0366-6999.213409 PMC5586193

[18] Feng Y, Zheng S, Sun Y, Liu L. Location-specific analysis of clinicopathological characteristics and long-term prognosis of primary Gastrointestinal diffuse large B-cell lymphoma. Sci Rep. 2025;15:19574. 40467905 10.1038/s41598-025-04537-9 PMC12137816

[19] Liu X, Lv T, Zhang X, Li J. Extensive small intestinal diffuse large B cell lymphoma. Rev Esp Enferm Dig. 2023;115:267. 36043558 10.17235/reed.2022.9100/2022

[20] Gupta V, Singh V, Bajwa R, Meghal T, Sen S, Greenberg D, Anne M, Levitt MJ. Site-Specific survival of extra nodal diffuse large B-Cell lymphoma and comparison with Gastrointestinal diffuse large B-Cell lymphoma. J Hematol. 2022;11:45–54. 35573751 10.14740/jh984 PMC9076143

[21] Halcu G, Evsei-Seceleanu A, Tapoi DA, Cerbu M, Barta C, Ceausu MC. Correlations between immunophenotypic markers and clinical progression in Romanian patients diagnosed with diffuse large B-Cell lymphoma. Med (Kaunas) 2025, 61. 10.3390/medicina61060948 PMC12194904 40572636

[22] Dickson BC, Serra S, Chetty R. Primary Gastrointestinal tract lymphoma: diagnosis and management of common neoplasms. Expert Rev Anticancer Ther. 2006;6:1609–28. 17134365 10.1586/14737140.6.11.1609

